# Saving and Empowering Young Lives in Europe (SEYLE): a randomized controlled trial

**DOI:** 10.1186/1471-2458-10-192

**Published:** 2010-04-13

**Authors:** Danuta Wasserman, Vladimir Carli, Camilla Wasserman, Alan Apter, Judit Balazs, Julia Bobes, Renata Bracale, Romuald Brunner, Cendrine Bursztein-Lipsicas, Paul Corcoran, Doina Cosman, Tony Durkee, Dana Feldman, Julia Gadoros, Francis Guillemin, Christian Haring, Jean-Pierre Kahn, Michael Kaess, Helen Keeley, Dragan Marusic, Bogdan Nemes, Vita Postuvan, Stella Reiter-Theil, Franz Resch, Pilar Sáiz, Marco Sarchiapone, Merike Sisask, Airi Varnik, Christina W Hoven

**Affiliations:** 1 National Swedish Prevention of Mental Ill-Health and Suicide (NASP), Karolinska Institutet, Stockholm, Sweden; 2 Feinberg Child Study Center, Schneider Children's Medical Center, Tel Aviv University, Tel Aviv, Israel; 3 Vadaskert Child and Adolescent Psychiatric Hospital, Budapest, Hungary; 4 Department of Psychiatry, School of Medicine, University of Oviedo; Centro de Investigación Biomédica en Red de Salud Mental, CIBERSAM. Oviedo, Spain; 5 Clinic of Child and Adolescent Psychiatry, University of Heidelberg, Heidelberg, Germany; 6 National Suicide Research Foundation, Cork, Ireland; 7 Clinical Psychology Department, Iuliu Hatieganu University of Medicine and Pharmacy, Cluj-Napoca, Romania; 8 Centre d'Epidémiologie Clinique -Inserm-EC CIE6, CHU de NANCY, hôpital Marin, Université H. Poincaré, Nancy, France; 9 Department of Psychiatry, Centre Hospitalo-Universitaire CHU de NANCY, Université H. Poincaré, Nancy, France; 10 Research Division for Mental Health, University for Medical Information Technology (UMIT), Innsbruck, Austria; 11 Mental Health Department, PINT, University of Primorska, Primorska, Slovenia; 12 Department of Medical and Health Ethics, Medical Faculty, University Hospital Basel, Basel, Switzerland; 13 Department of Health Sciences, University of Molise, Campobasso, Italy; 14 Estonian-Swedish Mental Health & Suicidology Institute, Tallinn, Estonia; 15 Department of Child and Adolescent Psychiatry, New York State Psychiatric Institute, Columbia University, New York, USA

## Abstract

**Background:**

There have been only a few reports illustrating the moderate effectiveness of suicide-preventive interventions in reducing suicidal behavior, and, in most of those studies, the target populations were primarily adults, whereas few focused on adolescents. Essentially, there have been no randomized controlled studies comparing the efficacy, cost-effectiveness and cultural adaptability of suicide-prevention strategies in schools. There is also a lack of information on whether suicide-preventive interventions can, in addition to preventing suicide, reduce risk behaviors and promote healthier ones as well as improve young people's mental health.

The aim of the SEYLE project, which is funded by the European Union under the Seventh Framework Health Program, is to address these issues by collecting baseline and follow-up data on health and well-being among European adolescents and compiling an epidemiological database; testing, in a randomized controlled trial, three different suicide-preventive interventions; evaluating the outcome of each intervention in comparison with a control group from a multidisciplinary perspective; as well as recommending culturally adjusted models for promoting mental health and preventing suicidal behaviors.

**Methods and design:**

The study comprises 11,000 adolescents emitted from randomized schools in 11 European countries: *Austria, Estonia, France, Germany, Hungary, Ireland, Israel, Italy, Romania, Slovenia and Spain*, with *Sweden *serving as the scientific coordinating center. Each country performs three active interventions and one minimal intervention as a control group. The active interventions include gatekeeper training (QPR), awareness training on mental health promotion for adolescents, and screening for at-risk adolescents by health professionals. Structured questionnaires are utilized at baseline, 3- and 12-month follow-ups in order to assess changes.

**Discussion:**

Although it has been reported that suicide-preventive interventions can be effective in decreasing suicidal behavior, well-documented and randomized studies are lacking. The effects of such interventions in terms of combating unhealthy lifestyles in young people, which often characterize suicidal individuals, have never been reported. We know that unhealthy and risk-taking behaviors are detrimental to individuals' current and future health. It is, therefore, crucial to test well-designed, longitudinal mental health-promoting and suicide-preventive interventions by evaluating the implications of such activities for reducing unhealthy and risk behaviors while concurrently promoting healthy ones.

**Trial registration:**

The German Clinical Trials Register, DRKS00000214.

## Background

Suicide is one of the leading causes of death worldwide, and the third leading cause of death among people aged below 25. Globally, every year, there are nearly a million deaths from suicide -- roughly one every 40 seconds [1,2]. Each year, in the 27 EU member states, approximately 63,000 Europeans commit suicide [3]; and, in 2006, suicide mortality exceeded the number of deaths due to traffic accidents [4]. Europe currently includes seven countries among the top 15 with the highest suicide mortality rates worldwide [5]. Moreover, among the 15-24 age group, it is estimated that approximately 100 to 200 suicide attempts take place for every completed suicide [6]. Research has demonstrated that suicidal behaviors are underestimated [2,7]: the actual prevalence of suicidal behavior is much higher than the reported rate. Unfortunately, comprehensive knowledge of the many risk factors associated with suicidal behavior in young people is lacking. It is, therefore, essential for research to focus on understanding the multiple underlying factors that contribute to or prevent suicidal behavior.

Suicidal behavior does not consist of isolated acts. Rather, it is the outcome of a long process usually associated with a psychiatric disorder [8-11] that, in many cases, goes undiagnosed and untreated [12]. There is, thus, evidence that suicidal behavior coincides with many underlying psychological and psychiatric conditions, ranging from depressive episode [13], anxiety [14] and alcoholism [15] to psychotic manifestations [16]. Psychological factors, though substantially interrelated with suicidal behaviors, are far from being the sole causes. In addition to psychiatric illnesses, certain risk behaviors have also been identified. For example, suicidal behaviors have been shown to be strongly associated with various types of risk behaviors, including peer victimization [17-19], risky sexual behavior [20], delinquency [21], substance abuse [22], non-suicidal self-injury (NSSI) [23], physical inactivity [24,25] and poor nutrition [26]. Risk behaviors rarely occur in isolation; rather, they tend to be integrated and often overlap in what is known as a '*risk behavior syndrome'*. Studies have demonstrated that risk behaviors are significantly correlated with one another and often appear in clusters [27-30]. Since unhealthy behaviors are significant predictors of subsequent mental health problems, and often occur in clusters, there is a paramount need to promote the adoption of healthy and positive lifestyles, especially during the early years of life.

Where unhealthy and risky behaviors are established in adolescence, the risk of health problems in adulthood is elevated. The association of such behaviors, with the leading causes of mortality and morbidity, underscores the importance of carrying out preventive interventions, particularly among young people [31], for the purpose of modeling healthy behaviors.

Effective prevention strategies should comprise measures that specifically focus on defined target groups. They should include evidence-based efforts designed to address an immediate problem, and, its underlying factors, through long-term follow-up. Accordingly, those few suicide prevention studies, which have been pursued among young people have included (i) gatekeeper training programs in schools [32] (ii) awareness-raising training among school pupils [33], combination of both [34], and (iii) professional screening [12,35,36] with subsequent clinical referral [37].

There is an ongoing debate in the scientific community about which strategy represents the most effective and efficient approach [38]. Reports indicate that suicide-preventive interventions in adults can reduce suicidal behavior [38,39], but well-documented and randomized studies for young people are still lacking.

The SEYLE (Saving and Empowering Young Lives in Europe) longitudinal research project is, therefore, based on a multi-site mental health promotion and suicide prevention program; studying the three above-mentioned strategies separately to understand which approach is the most effective and pragmatic across the participating schools, and considers cultural and national differences; as well as recommending evidence-based, combined and multifaceted interventions.

## Objectives

The key objectives of the study are:

(i) to collect baseline and follow-up assessments of the mental health and well-being, alongside demographic data, information about lifestyles, values, risk behaviors and other psychosocial information of European adolescents and compile an epidemiological database;

(ii) to carry out an evaluation of three types of interventions: gatekeeper training involving referrals by teacher and school staff, awareness-raising training for pupils encouraging self-referral and professional screening with subsequent clinical referral among adolescents; in comparison with a control group that comprises self-referral;

(iii) to focus on reducing risk-taking and suicidal behavior while simultaneously promoting improved mental health;

(iv) to evaluate the intervention outcomes (in terms of the efficacy, maintenance, effectiveness and cost-effectiveness of the programs), in a multidisciplinary (i.e. social, psychological and economic) perspective, in comparison with a control group;

(v) to evaluate treatment and social support outcomes for referred pupils.

## Methods

### Study design

The study is a randomized controlled trial (registered in the German Clinical Trials Register, DRKS00000214) that assesses three different types of intervention strategies in comparison with a control group. Using a factorial design, the study estimates and compares the effects of different suicide-prevention programs on unhealthy lifestyles, in the form of risk and suicidal behaviors (Table 1).

**Table 1:** Factorial design of interventions

ARM (*n = 250 subjects per arm in each country*)	Gatekeeper Training (QPR)	Awareness training	Professional Screening
I	X		
II		X	

III			X

IV	Control Group/Minimal Intervention		

**Figure 1:**
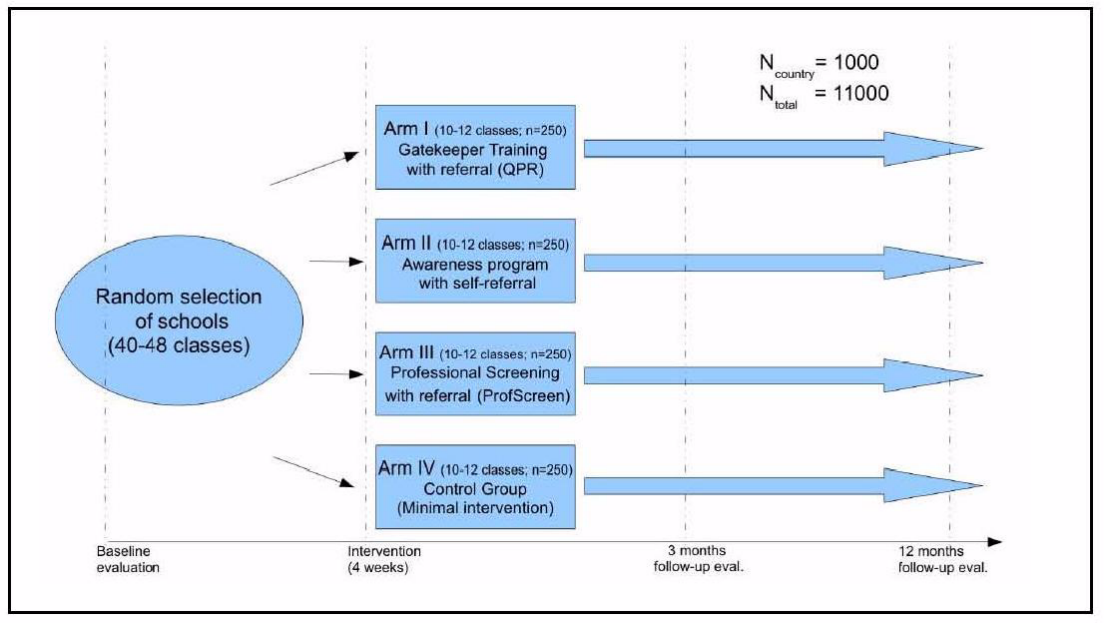
**General study design of SEYLE**.

This 12-country study comprises a random selection of schools in 11 European countries, including *Austria, Estonia, France, Germany, Hungary, Ireland, Israel, Italy, Romania, Slovenia and Spain*, with *Sweden *serving as the scientific coordinating center. The interventions are implemented in the school premises and coordinated by each country's respective SEYLE center. The general study design of SEYLE is illustrated in Figure 1.

### Population and sampling procedures

The target sample for each intervention 'arm' as well as for the control 'arm' is 250 pupils, i.e. 1,000 subjects in each participating country (totaling 11,000 subjects overall).

In each study site, a catchment area is identified and a list of eligible schools generated. Eligible schools are categorized by size as (1) **small **(less than or equal to the median number of pupils in all schools in the study catchment area or region) and (2) **large **(greater than the median number of pupils in all schools in the study catchment area or region). Every class in each school selected (regardless of size) where 15-year-old pupils make up a majority is surveyed. This age group is selected because of its risk propensity and the feasibility of performing 12-month follow-ups. Schools are randomized on the basis of their size category and sequentially assigned to respective intervention and control arms, comprising both large and small schools. The remaining large and small schools are then sequentially numbered.

To avoid contamination and confounding, only one type of intervention is performed in each school. Given the insufficient evidence of effectiveness of the interventions, equipoise can be assumed so that no institution or group will be put at (dis)advantage systematically. Schools are only aware of the respective intervention arm implemented at their facility, i.e. pupils are not informed of the other types of intervention performed in other schools. The effect that information could eventually spread through informal suggestions can be neglected; in case this becomes a topic, project members would apply a strategy to openly give appropriate additional information. A coordinator is assigned to each intervention arm and its implementation. Coordinators in the respective schools for each arm are instructed only on how to implement their own intervention arm, and have no prior experience of the procedures for the other interventions. Informed consent to participate in the study is obtained from all the adolescents and their parents.

### Inclusion and exclusion criteria

Schools and adolescents in the study areas are eligible to participate if they meet all the following criteria:

(1) the school authority agrees to participate;

(2) the adolescents attend non-specialist public schools;

(3) school contains at least 40 pupils aged 15;

(4) school has more than two (3+) teachers for pupils aged 15;

(5) no more than 60% of pupils are of either sex;

(6) informed consent from parents and pupils is obtained.

If the school-based adolescents meet the following exclusion criteria, they are ineligible to participate:

(1) the school authority refuses to participate;

(2) the adolescents attend a specialist and/or independent or private school;

(3) the adolescents attend single-sex schools;

(4) a school has fewer than 40 pupils aged 15;

(5) the parents of pupils in a participating school, or the pupils themselves, have refused to sign the consent document.

### Identification of referral facilities

In the SEYLE project, healthcare facilities that are available to receive the referral of pupils and provide treatment are identified within each respective community prior to the commencement of the project. Pupils who are categorized as high risk for mental ill-health or suicidal behavior are remitted to the local healthcare facilities for professional treatment. Pupils who do not meet the criteria of high risk for mental illness or suicidal behaviors, but necessitate changing or improving their lifestyles, are referred to a non-clinical healthy lifestyle group for social support and development.

#### Healthcare services

Prior to the launch of the SEYLE project, all local healthcare services in each respective center are identified, including general practitioners, public healthcare facilities and specialized psychiatrists and psychologists. Personnel is informed about the project and notified regarding the possibility of subsequent increases of pupil referrals. Information describing the SEYLE project is provided to all local healthcare services, including contact information for SEYLE researchers, and information on suicide prevention interventions [40,41]. All adolescents ascertained to be at-risk are referred by professionals, or self-referred, to the local healthcare facilities for treatment.

#### Healthy Lifestyle Group

Pupils who are referred by teachers, or by themselves, for perceiving to have at-risk behaviors, but who are not in need of professional help, are recommended to a non-clinical healthy lifestyle group. The healthy lifestyle groups comprise facilities in which pupils are positively encouraged to adopt or improve healthy behaviors. On the local level, this could be a boy scouts club, organized sport activities and other local activities in the community. On the national level, healthy lifestyle groups could be national adolescent self-help programs, etc. Moreover, SEYLE centers unable to identify sufficient healthy lifestyle groups are encouraged to create their own version of a healthy lifestyle group in which they choose the topics and involve local volunteers to organize the meetings. The concept of the healthy lifestyle group is to provide a positive and uplifting localized atmosphere for adolescents who are not classified as high risk and do not fit the criteria for professional help; however, do need positive support for adopting healthy behaviors and changing unhealthy ones.

### Baseline assessment of pupils

The baseline evaluation questionnaire, completed within the confines of the classroom, is followed up with a post-intervention evaluation questionnaire 3- and 12-months post-baseline to study changes in attitudes, lifestyles, behaviors and mental health problems of pupils. The baseline assessment obtains data on lifestyles, behaviors, values, mental health and suicidality. Data are collected by means of structured questionnaires, including:

(i) the *Global School-Based Pupil Health Survey *(GSHS) [42], which assesses lifestyles and risk-taking behaviors;

(ii) the *WHO Well-being Scale *(WHO-5) [43], which evaluates mood (good spirits, relaxation), vitality (being active and waking up fresh and rested) and general interests (being interested in things);

(iii) the *Beck Depression Inventory *(BDI) [44], which measures depressive symptoms;

(iv) the *Paykel Suicide Scale *(PSS) [45], which determines suicidal ideation and suicidal behavior;

(v) the *Strengths and Difficulties Questionnaire *(SDQ) [46], which collects information on emotional symptoms, conduct problems, hyperactivity and/or inattention, peer relationship problems and pro-social behavior;

(vi) the *Deliberate Self-Harm Inventory *(DSHI) [47], which evaluates deliberate self-harm behavior;

(vii) the *Young's Diagnostic Questionnaire *(YDQ) [48] for Internet Addiction, which identifies Internet dependency among adolescents;

(viii) questions from the *European Values Study *(EVS) [49], which examines values, such as religion, family, marriage, work and friendship;

(ix) specific items developed or modified for the SEYLE study, concerning reading, music, and internet habits, as well as coping, trauma and bullying, stressful life events, stigma and discrimination, peer and parent-child relations, children's physical health, alcohol and substance use, and future outlook.

### Emergency cases

A specific procedure to evaluate and immediately assist emergency cases is compulsory for all pupil participation of the SEYLE project. Emergency cases are identified by means of two specific questions prompted in the baseline questionnaire. Pupils are considered emergency cases if they respond "sometimes", "often", "very often" or "always" to the question "*During the past two weeks, have you reached the point where you seriously considered taking your life or perhaps made plans how you would go about doing it*?"; and/or if they respond "Yes" to the question "*Have you tried to take your own life during the past 2 weeks*?". Pupils identified in the baseline questionnaire as emergency cases are immediately referred for clinical evaluation and directed to healthcare services for treatment if necessary. However, once evaluated, and even when subjected to treatment, pupils are permitted to continue in the intervention arm to which they were originally assigned.

### Interventions

The preventive interventions comprise: Gatekeeper Training (QPR), training of pupils in awareness of mental health and crisis management (Awareness Training), and screening of at-risk pupils by health professionals (Professional Screening) with subsequent clinical evaluation. These three types of intervention arms are compared with the control group. Interventions are designed to promote overall healthy behaviors; raise awareness; improve lifestyles; refer subjects who demonstrate signs of suicidal risk and mental ill-health for treatment or to a non-clinical healthy-lifestyle group; and ultimately, enhance psychological well-being while reducing suicidal risk and mental illness.

#### I. Question, Persuade and Refer (QPR)

The QPR 'preventive intervention' program, developed in the US http://www.qprinstitute.com/, focuses primarily on training gatekeepers to identify and intervene when individuals are engaged in risk behaviors. It involves asking the individuals questions concerning their behavior, *persuading *them to seek help if they are displaying suicidal warning signs and, when appropriate, *referring *the individual to a treatment facility. In medical ethics, the doctrine of Informed Consent and respecting the individual's rights does not preclude persuasion [50,51]. Gatekeepers, in this study, are teachers and school staff who are in daily contact with the subjects concerned. Teachers and school staff in the randomly selected schools are trained by staff in the SEYLE project that have undergone the official QPR training program in the USA, or online, and are certified trainers of this method. Training consists of a two-hour interactive lecture and a one-hour role-play session. Teachers and school staff receive a QPR booklet on suicide prevention with education that focuses on describing the epidemiology and risk factors of the phenomenon of suicide; deals with common myths and facts about suicide; provides detailed guidance on how to recognize young people at-risk; and gives basic information about how to support pupils who are contemplating suicide and persuade them to get help. SEYLE has, however, modified one aspect of the QPR intervention in order to fit the needs of the project. In the original QPR intervention, business cards with information concerning contact information for local healthcare services are distributed to the gatekeepers during the training, in which case, gatekeepers keep the business cards on their person in the occurrence they need to utilize the information when referring someone presumed to be at-risk.

In the SEYLE modified version, the business cards contain contact information not only for healthcare services, but for non-clinical healthy lifestyle groups as well. Moreover, business cards are dispersed to each teacher and school staff participant during the training advising them to distribute the business cards to adolescents who they presume to be at-risk for mental ill-health or suicidal behavior.

The active intervention period for the QPR in SEYLE is a period of four weeks.

#### II. Awareness Training of Pupils

The awareness intervention is designed to promote knowledge of mental health, healthy lifestyles and behaviors among adolescents enrolled in the SEYLE project. It is an extended, refined version of an awareness trial conducted in nine countries [33] developed by researchers from Columbia University, New York and the National Prevention of Suicide and Mental Ill-Health (NASP), Karolinska Institutet, Sweden and incorporates methodology used in preventive interventions for suicidal behavior [52]. All pupils in the schools concerned are provided with a customized educational, awareness-raising booklet covering six specific topics concerning: (i) awareness of mental health; (ii) self-help advice; (iii) stress and crisis; (iv) depression and suicidal thoughts; (v) helping a troubled friend; and (vi) getting advice - who to contact [53,54] with telephone numbers and email addresses to local healthcare facilities and healthy lifestyle groups in case pupils wish to seek help. Once the intervention commences, six posters are hung in the classroom covering the six key topics as in the awareness booklets. Lessons, which are also combined with role-play sessions, address the six topics covered in the awareness booklet and posters.

**Figure 2:**
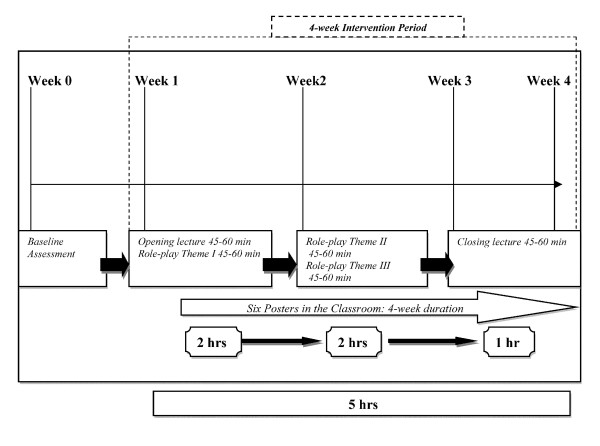
**Timeline for the Awareness Intervention**.

During the classroom sessions, the instructor and an assistant distribute the awareness booklets to all the pupils. The instructor addresses these six topics along with role-play sessions during subsequent five one-hour sessions over 4-week duration (Figure 2).

In the role-play sessions, the adolescents have the opportunity to act out conflict issues they experience in their everyday lives (i.e. with parents, peers, teachers etc.) under the supervision of the same trained instructor who gives the lectures and leads role-play sessions, along with an assistant, while pupils acquire skills in resolving such problems. The role-play sessions comprise the following three themes: **Theme I**, *Awareness about choices*; **Theme 2**, *Awareness about feelings and how to manage stress and crisis situations*; and **Theme 3**, *Awareness about depression and suicidal thoughts*. Pupils who, through this intervention, recognize their own need for help have the opportunity and are encouraged to self-refer themselves to contact an appropriate mental-healthcare provider, or join a healthy lifestyle group by using the country-specific contact information that is provided in the booklets and on a business card, which is distributed to each pupil.

#### III. Professional Screening

This intervention is designed to help health professionals to identify at-risk adolescents by using cut-off points for positive responses based on specific scales of adolescent mental health in the baseline questionnaire. This intervention was developed by the University of Heidelberg, a SEYLE center, and NASP at Karolinska Institutet, the coordinating center, and pilot-tested in the Heidelberg clinic. Based on the results of the pilot test, cut-off points were assigned accordingly (*see *Table 2). Pupils who screen at or above specific cut-off points are referred for professional clinical assessment. This assessment is conducted by a psychiatrist or clinical psychologist, who performs a semi-structured clinical interview designed for the evaluation of mental health problems, as well as self-destructive and risk-taking behaviors for adolescents screened as 'at-risk' in the baseline evaluation in accordance to the cut-off criteria.

**Table 2:** Cut-off criteria in the baseline questionnaire and in the professional screening intervention for selected at-risk pupils referral to clinical assessment

Theme		Cut-off value/threshold value	Risky and self-injurious behavior is diagnosed when
**Depression (BDI)**		BDI-score ≥ 14; depending on the responses, from 0 to 3 points are assigned (cf. manual) and added.	A BDI score of ≥ 14 is obtained.

**Anxiety (ZUNG)**		ZUNG-score ≥ 45; depending on the responses, from 1 to 4 points are assigned and added.	A ZUNG score of ≥ 45 is obtained.

**Suicidal Ideation and Attempts**		PAYKEL Scale	The cut-off of at least one single item is obtained.
	
		Yes/No response: previous suicide attempt.	'Yes' is the response given.

**Non-suicidal self-injury**		Deliberate Self-Harm Inventory (DSHI)	A sum of ≥ 2 is obtained and all points must therefore be added.

**Eating behavior**		Both responses are needed to calculate the BMI score.	The BMI score is less than 16.5.

**Sensation-seeking and delinquent behaviors**		Yes/No response: riding with someone who has been drinking.	The sum of ≥ 3 for the theme 'risk behavior' is obtained. All points must therefore be added.
			
		Yes/No response: skateboarding or riding roller-blades in traffic and without a helmet.	
			
		Yes/No response: subway cart jumping, or held on the back of a moving vehicle.	
			
		Yes/No response: visiting known areas that are dangerous during night.	
			
		Sexual Promiscuity Unprotected Sex	

**Substance abuse**	**Tobacco**	Tobacco Use (lifetime measure)	'Yes' is the response given to tobacco use, **and **2 cigarettes per day or more for tobacco consumption frequency.
			
		Tobacco Consumption Frequency	
	
	**Alcohol**	Alcohol Consumption Frequency (12-month measure)	2 times per week or more
		
		Alcohol Consumption Amount (12-month measure)	3 or more drinks in a typical drinking day
		
		Alcohol Intoxication (lifetime measure)	3 times or more
		
		Alcohol Hangover (lifetime measure)	3 times or more
	
	**Illegal drugs**	Illicit Drug Consumption (lifetime measure)	3 times or more

**Exposure to media**		Media Exposure Frequency	Option 4, 5 or 6 is ticked, i.e. a pupil spends at least 'five to six hours per day' watching television, playing computer games etc.

**Social relationships**		Loneliness Frequency (12-month measure)	Option 4 ('most of the time') or 5 ('always') is checked.

**Bullying**		Peer Victimization (12-month measure)	The sum of ≥ 5 is obtained. All response options must therefore be added.

***School attendance***		Truancy (2-week measure)	Option 3, 4 or 5 is ticked, i.e. respondents have missed three or more days of school or class without permission.

The time period for the active intervention in the Professional Screening arm is 4-week duration.

All pupils with a predetermined cut-off for depression, anxiety, phobia, alcoholism, substance abuse, non-suicidal self-injury (NSSI) and suicidality are referred for professional treatment. Pupils with social problems are referred to an appropriate non-clinical healthy-lifestyle group.

#### IV. Control group/Minimal Intervention

For ethical reasons (nonmaleficence/preventing harm; fairness/equitable access), the control group cannot be completely excluded from any intervention [55]. Therefore, a minimal intervention comprising six educational posters, which are the same as those utilized in the awareness training intervention (*see above*), are displayed in the classrooms. The posters display six key points, the same as in the awareness arm booklet, and provide contact details for the local healthcare services and healthy lifestyle groups. Pupils who recognize their own need for help have the opportunity to contact (self-referral) healthcare providers or a healthy lifestyle group. This minimal intervention for the control group includes no other form of intercession.

The posters hang in the classroom for four weeks, as all interventions performed in SEYLE have an active intervention period of 4 weeks.

### Pupil referrals in each intervention

During and after the SEYLE interventions, students at-risk are actively referred to local health-care facilities and to healthy lifestyle groups. Students are referred according to the arm they were randomized to. In the QPR arm, teachers and school staff refer pupils; in the Awareness and Control arms, pupils self-refer; and in the Professional Screening arm, the healthcare professional refers the pupils. Pupil consignment is based on the level of risk for each pupil.

### 3- and 12-month follow-up assessment for pupils

The assessment instruments used for the baseline measurement (GSHS, WHO-5, PSS, SDQ, BDI, DSHI, EVS questions and SEYLE-specific questions) are also used for the 3- and 12-month follow-up evaluations. These measures cover the same outcome variables as those in the baseline assessment in order to investigate changes. The follow-up questionnaire also includes key questions covering information on the use of referrals by teachers, school staff, health professionals and self-referrals. The follow-up assessment comprises the description of treatment received, as well as an evaluation of the intervention study activities performed by teachers, school staff and health professionals.

### Outcome measures

Outcome variables that are assessed in the project include well-being, depression, anxiety, emotional and conduct problems, coping, self-destructive and addictive behaviors, values, and lifestyles. Table 3 illustrates the outcome variables and the corresponding assessment tools utilized to measure them.

**Table 3:** Correspondence between questionnaire measures and study outcomes

Tool for measurement	Outcome variables
WHO-5	General well-being

Beck Depression Inventory (BDI)	Depression

Paykel Suicide Scale (PSS)	Suicidal behavior

Global School-Based Pupil Health Survey (GSHS)	Alcohol use and abuse
	
	Drug use and abuse
	
	Eating habits
	
	BMI
	
	Physical activity
	
	Sexual habits
	
	Tobacco use
	
	Violent behaviors
	
	Risky behaviors

Strengths and Difficulties Questionnaire (SDQ)	Emotional symptoms
	
	Conduct problems
	
	Hyperactivity/inattention
	
	Peer relationship problems
	
	Pro-social behavior

European Values Study Questionnaire (EVS)	Values (religion, family, marriage, work, friendship)

Specific SEYLE questions	Coping
	
	General child health
	
	Peer relations
	
	Child-parent relations
	
	Stigma and discrimination
	
	Future outlook

Deliberate Self Harm Inventory (DSHI)	Self-harm behavior

Young's Diagnostic Questionnaire (YDQ) for Internet Addiction	Internet addictive behavior

Another outcome variable is pupil referrals, i.e. the total number of referrals inclusive all emergency cases identified during the baseline evaluation, and treatment outcomes. For data collection, SEYLE has developed a systematic method of recording and monitoring all referrals and obtaining feedback on their appropriateness. Pupils are asked whether they have been referred and to whom, what kinds of treatment they have received (medication, psychotherapy, both or neither etc.) and for how long. Phone calls are performed with pupils who do not participate in the follow-up evaluations, and, where possible, facilitators maintain contact with the pupils' parents. In cases, where parents or family represent a source of concern in the perception of the pupil or staff member, contacts will be handled in a particularly careful manner [56].

### Professionals, teachers and school staff assessment

Baseline and 3- and 12-month evaluations is also performed among health professionals, teachers and school staff involved in the project. Health professionals are assessed by a short 12-item questionnaire on their knowledge and preparedness of treating adolescents displaying suicidal behaviors. Teachers and school staff undergo a more detailed assessment questionnaire that collects data on mental health and suicidal behavioral knowledge, perception and attitudes towards mental health and suicide, employment satisfaction, their personal well-being and perspective of the SEYLE project.

### Data analysis

The SEYLE project generates a total sample of 11,000 European adolescents, with 8,250 (750 per site) receiving one or other of the three interventions being tested. The control arm contributes 2,750 adolescents (250 per site) to the total sample.

Power calculations adhere to the widely accepted proposals made by Cohen (1988) [57] for detection of small, medium and large effects. For all outcome measures, the sample size gives the study more than 80% statistical power to detect medium effects within the individual centers and small effects at the aggregate level of centers. Overall, the SEYLE intervention project is expected to show medium effect changes.

The SEYLE study sample potentially exceeds the sample size requirements in order to detect statistically significant changes. This will ensure the required statistical power, taking into account the possibility of some center recruiting fewer pupils than expected, attrition rates at follow-up and missing data. An initial stage of statistical analysis involves examining the consistency of psychometric properties across sites of the measures used in the SEYLE study. Reliability analysis is performed on the relevant data from each participating center. The suitability of continuous variables for parametric tests is assessed.

In cases where the diagnostics indicate that the reliability of the parametric tests may be significantly undermined, the appropriate non-parametric test is carried out. These include the Mann-Whitney test, the Kruskal-Wallis test, the Wilcoxon test and Friedman's ANOVA. Comparisons between study arms in relation to dichotomous and polychotomous variables are initially made using Fisher's exact test and chi-square tests, as appropriate. Logistic regression compares the intervention arms to the control arm in relation to the risk of an event of interest occurring in the follow-up period. The odds ratio, with its 95% confidence interval, is used as the measure of relative risk. An adjusted odds ratio is produced from multivariate logistic regression models, which include relevant covariates. Statistical analyses are carried out at the level of the individual centers and at the aggregate level. Variation in the experimental effects is examined across the 11 participating centers.

### Research Ethics

The study was approved ethically by the European Commission as a precondition of funding approval for the project. Ethical permission for the project, including permission to follow up individual pupils, has also been obtained in each participating country by the Research Ethics Committees. All requirements of obtaining Informed Consent from pupils and parents are followed carefully. In order to maintain confidentiality and to allow for analyzing follow-up data in the individual, questionnaires include a specific code to identify each participating pupil, enabling data to be obtained at individual and not only aggregate level. An independent ethical advisor supervises the implementation of the ongoing project in order to ensure maximum protection of vulnerable individuals such as adolescents and articulate any sensitive issues [58].

## Discussion

The three prevention strategies that are tested in SEYLE are built upon the concept of empowering different key persons. Each prevention strategy is governed by different scientific perspectives of empowerment.

The first strategy, gatekeeper training, encompasses education concerning mental health and suicidal behavior for key persons or 'gatekeepers', i.e. persons in frequent contact with adolescents such as teachers and school staff. Through this training, the gatekeepers learn how to persuade at-risk adolescents to seek clinical help, which essentially empowers the 'gatekeeper'. This strategy has been moderately successful [32,59-62].

The second strategy, awareness-raising training, involves interactively teaching school pupils the importance of mental health. Consequently, it empowers individuals to identify their personal level of risk, as well as that of their peers, while informing them how best to seek appropriate care, and, if necessary, helping them to do so.

Finally, professional screening with subsequent clinical referral is an approach designed to evaluate a specific target group by utilizing a well-structured assessment instrument based on cut-off scores for meeting certain criteria for mental health problems. Individuals meeting these criteria are referred for clinical evaluation, if necessary, with appropriate treatment determined by the professional in charge. This strategy empowers the professional involved in the screening.

To date, the effects of suicide-preventive interventions in young people in terms of improving unhealthy lifestyles have not yet been reported. We know that unhealthy and risk-taking behaviors are detrimental to one's current and future health. For a number of disorders and illnesses, they are important factors contributing to premature mortality and morbidity. These types of behavior may be expected to be modifiable and even preventable with appropriate intervention measures. It is, therefore, crucial to test well-designed, longitudinal health-promoting and suicide-preventive interventions by evaluating to what extent such activities reduce unhealthy behaviors while simultaneously promoting healthy ones. The SEYLE project is unique in this respect, since suicide-preventive interventions have not previously been tested with long-term follow-up measures to assess changes in unhealthy behaviors.

The strength of SEYLE in comparison with other school-based prevention and health promotion programs is the active referral of all emergency cases to professionals. According to Mann et al. [38], prevention programs for children and adolescents, such as curriculum-based programs, have shown mixed results in terms of effectiveness and impact. Knowledge about suicide has improved, but there have been both beneficial and harmful effects in terms of help-seeking, attitudes and peer support. Curriculum-based programs increase knowledge and improve attitudes concerning mental illness and suicide, but the evidence that they prevent suicidal behavior is insufficient [63]. Such programs may even be detrimental for emergency cases or high-risk pupils, if they do not provide direct access to care [63]. This risk will be systematically prevented in SEYLE. Moreover, psychiatric and psychological treatment are preferred options for pupils who are identified as high risk; however, some pupils may not fit the criteria to receive professional treatment, thus, it is of interest to examine the effectiveness of healthy lifestyle groups for those particular adolescents.

There are also some limitations of the study. Some families may pose problems to allow for an informed consent of an adolescent child to join the project. This may be related to dysfunctional processes in the family affecting the child's health [64]. In the SEYLE project, due to economical limitations, we are unable to examine the source of such family conflicts and, as a result, it can cause some selection bias of pupils joining the interventions. Other limitations of the study include pupils' refusal to partake in the referral process to healthcare facilities or follow-up evaluations in all intervention arms. Moreover, the information collected on treatment for pupils referred to healthcare services and healthy lifestyle groups is based on self-reports by the pupil, and is not collected from medical records or from leaders in the healthy lifestyle groups, however, in respective centers, this option is a possibility and data is collected from medical records wherever possible.

In conclusion, the proposed pragmatic SEYLE trial is expected to provide scientific evidence for understanding the effects of different preventive interventions, their cost-effectiveness and how they can also be combined and practically utilized.

## Ethical approval

The SEYLE protocol has been granted ethical approval in each participating country where the research project is implemented:

• **Austria**: Ethikkomission der Medizinischen Universität Innsbruck

• **Estonia**: Tallinna Meditsiiniuuringute Eetikakomitee

• **France**: Comité de Protection des Personnes Sud-Méditerranée II

• **Germany**: Ethikkommission Medizinische Fakultät Heidelberg

• **Hungary**: Egészségügyi Tudományos Tanács Titkárság, Pályázati Iroda, Tudományos És Kutatásetikai Bizottság

• **Ireland**: Clinical Research Ethics Committee of the Cork Teaching Hospital

• **Israel**: Helsinki Committee at the Rabin Medical Center

• **Italy**: Comitato Bioetico Di Ateneo, Università Degli Studi Del Molise

• **Romania**: Comisia De Eticã, A Universitãtii De Medicinã Si Farmacie, Cluj Napoca

• **Slovenia**: Komisija Republike Slovenije Za Medicinsko Etiko

• **Spain**: Comité Ètico de Investigación Clinica, regional del Principado de Asturias

## Competing interests

The authors declare that they have no competing interests.

## Authors' contributions

DW is the principal investigator, participated in the study design and coordination, and critically revised all the phases of the manuscript. VC participated in the study design and coordination, co-drafted the manuscript, and participated in the critical revision of the manuscript. MS participated in the design of the study and coordination, and critically revised the manuscript. TD participated in the coordination of the study, co-drafted the manuscript, and implemented all revisions to the manuscript. CW participated in the design of the study and coordination, provided consultation on anthropological issues, advised on research methodology, critically revised the manuscript, and drafted the final version of the manuscript. CH participated in the design of the study, provided consultation for epidemiological issues, advised on research methodology and critically revised the manuscript.

CH, AV, JPK, RB, JB, PC, AA, MS, DC, DM and JB are the principal investigators for the SEYLE project in their respective countries. SRT is the expert ethical advisor for the SEYLE project, providing consultation for the study design and ongoing interventions. The other authors are the site coordinators for the SEYLE center in their respective countries. All authors read and approved the final manuscript.

The Professional Screening Intervention was designed by the University of Heidelberg, RB, FR, MK, and NASP, DW and VC.

The Awareness Training Intervention was designed by Columbia University, CH, CW, and NASP, DW.

## Pre-publication history

The pre-publication history for this paper can be accessed here:

http://www.biomedcentral.com/1471-2458/10/192/prepub

## References

[B1] World Health Organization Self Directed Violence http://www.who.int/violence_injury_prevention/violence/global_campaign/en/chap7.pdf

[B2] Bertolote JM Fleischmann A Wasserman D, Wasserman C A global perspective on the magnitude of suicide mortality The Oxford Textbook of Suicidology and Suicide Prevention: A Global Perspective 2009 Oxford: Oxford University Press 91 98

[B3] Eurostat EUROSTAT Year Book 2009 http://epp.eurostat.ec.europa.eu/portal/page/portal/publications/eurostat_yearbook

[B4] Eurostat Statistics http://epp.eurostat.ec.europa.eu/portal/page/portal/eurostat/home/

[B5] Suicide rates http://www.who.int/mental_health/prevention/suicide_rates/en/

[B6] Bertolote JM Fleischmann A Wasserman D, Wasserman C Suicide thoughts, suicide plans and attempts in the general population on different continents The Oxford Textbook of Suicidology and Suicide Prevention: A Global Perspective 2009 Oxford: Oxford University Press 99 104

[B7] Miller M Azrael D Hemenway D The epidemiology of case fatality rates for suicide in the northeast Ann Emerg Med 2004 43 6 723 730 10.1016/j.annemergmed.2004.01.018 15159703

[B8] Brent DA Baugher M Bridge J Chen T Chiappetta L Age- and sex-related risk factors for adolescent suicide J Am Acad Child Adolesc Psychiatry 1999 38 12 1497 1505 10.1097/00004583-199912000-00010 10596249

[B9] Shaffer D Gould MS Fisher P Trautman P Moreau D Kleinman M Flory M Psychiatric diagnosis in child and adolescent suicide Arch Gen Psychiatry 1996 53 4 339 348 8634012 10.1001/archpsyc.1996.01830040075012

[B10] Cohen LJ Test MA Brown RL Suicide and schizophrenia: data from a prospective community treatment study Am J Psychiatry 1990 147 5 602 607 2327487 10.1176/ajp.147.5.602

[B11] Mittendorfer-Rutz E Rasmussen F Wasserman D Familial clustering of suicidal behaviour and psychopathology in young suicide attempters. A register-based nested case control study Soc Psychiatry Psychiatr Epidemiol 2008 43 1 28 36 10.1007/s00127-007-0266-0 17934681

[B12] Shaffer D Scott M Wilcox H Maslow C Hicks R Lucas CP Garfinkel R Greenwald S The Columbia Suicide Screen: validity and reliability of a screen for youth suicide and depression J Am Acad Child Adolesc Psychiatry 2004 43 1 71 79 10.1097/00004583-200401000-00016 14691362

[B13] Hollis C Depression, family environment, and adolescent suicidal behavior J Am Acad Child Adolesc Psychiatry 1996 35 5 622 630 10.1097/00004583-199605000-00017 8935209

[B14] D'Attilio JP Campbell B Relationship between death anxiety and suicide potential in an adolescent population Psychol Rep 1990 67 3 Pt 1 975 978 10.2466/PR0.67.7.975-978 2287690

[B15] Buri C von Bonin B Strik W Moggi F Predictors of attempted suicide among swiss patients with alcohol-use disorders J Stud Alcohol Drugs 2009 70 5 668 674 19737490 10.15288/jsad.2009.70.668

[B16] Nishida A Sasaki T Nishimura Y Tanii H Hara N Inoue K Yamada T Takami T Shimodera S Itokawa M Psychotic-like experiences are associated with suicidal feelings and deliberate self-harm behaviors in adolescents aged 12-15 years Acta Psychiatr Scand 2009 in press 19614622 10.1111/j.1600-0447.2009.01439.x

[B17] Brunstein Klomek A Marrocco F Kleinman M Schonfeld IS Gould MS Bullying, depression, and suicidality in adolescents J Am Acad Child Adolesc Psychiatry 2007 46 1 40 49 10.1097/01.chi.0000242237.84925.18 17195728

[B18] Klomek AB Sourander A Niemela S Kumpulainen K Piha J Tamminen T Almqvist F Gould MS Childhood bullying behaviors as a risk for suicide attempts and completed suicides: a population-based birth cohort study J Am Acad Child Adolesc Psychiatry 2009 48 3 254 261 10.1097/CHI.0b013e318196b91f 19169159

[B19] Kaminski JW Fang X Victimization by Peers and Adolescent Suicide in Three US Samples J Pediatr 2009 155 5 683 8 10.1016/j.jpeds.2009.04.061 19616788

[B20] Houck CD Hadley W Lescano CM Pugatch D Brown LK Suicide attempt and sexual risk behavior: relationship among adolescents Arch Suicide Res 2008 12 1 39 49 10.1080/13811110701800715 18240033

[B21] Brent DA Bridge JA Delinquent accounts: does delinquency account for suicidal behavior? J Adolesc Health 2007 40 3 204 205 10.1016/j.jadohealth.2006.12.014 17321419

[B22] Schneider B Kolves K Blettner M Wetterling T Schnabel A Varnik A Substance use disorders as risk factors for suicide in an Eastern and a Central European city (Tallinn and Frankfurt/Main) Psychiatry Res 2009 165 3 263 272 10.1016/j.psychres.2008.03.022 19185355

[B23] Brunner R Parzer P Haffner J Steen R Roos J Klett M Resch F Prevalence and psychological correlates of occasional and repetitive deliberate self-harm in adolescents Arch Pediatr Adolesc Med 2007 161 7 641 649 10.1001/archpedi.161.7.641 17606826

[B24] Brown DR Blanton CJ Physical activity, sports participation, and suicidal behavior among college students Med Sci Sports Exerc 2002 34 7 1087 1096 10.1097/00005768-200207000-00006 12131246

[B25] Brown DR Galuska DA Zhang J Eaton DK Fulton JE Lowry R Maynard LM Psychobiology and behavioral strategies. Physical activity, sport participation, and suicidal behavior: U.S. high school students Med Sci Sports Exerc 2007 39 12 2248 2257 10.1249/mss.0b013e31815793a3 18046198

[B26] Ahren-Moonga J Holmgren S von Knorring L Af Klinteberg B Personality traits and self-injurious behaviour in patients with eating disorders Eur Eat Disord Rev 2008 16 4 268 275 10.1002/erv.860 18240124

[B27] Flisher AJ Kramer RA Hoven CW King RA Bird HR Davies M Gould MS Greenwald S Lahey BB Regier DA Risk behavior in a community sample of children and adolescents J Am Acad Child Adolesc Psychiatry 2000 39 7 881 887 10.1097/00004583-200007000-00017 10892230

[B28] Roberfroid DPJ Psychosocial factors and multiple unhealthy behaviours in 25- to 64-year-old Belgian citizens Arch Public Health 2001 59 281 307

[B29] Winters KC Botzet AM Fahnhorst T Baumel L Lee S Impulsivity and its Relationship to Risky Sexual Behaviors and Drug Abuse J Child Adolesc Subst Abuse 2009 18 1 43 56 10.1080/15470650802541095 19777076 PMC2748350

[B30] King RA Schwab-Stone M Flisher AJ Greenwald S Kramer RA Goodman SH Lahey BB Shaffer D Gould MS Psychosocial and risk behavior correlates of youth suicide attempts and suicidal ideation J Am Acad Child Adolesc Psychiatry 2001 40 7 837 846 10.1097/00004583-200107000-00019 11437023

[B31] Millstein SG Irwin CE Jr Adler NE Cohn LD Kegeles SM Dolcini MM Health-risk behaviors and health concerns among young adolescents Pediatrics 1992 89 3 422 428 1741215

[B32] Wyman PA Brown CH Inman J Cross W Schmeelk-Cone K Guo J Pena JB Randomized trial of a gatekeeper program for suicide prevention: 1-year impact on secondary school staff J Consult Clin Psychol 2008 76 1 104 115 10.1037/0022-006X.76.1.104 18229988 PMC2771576

[B33] Hoven CW Wasserman D Wasserman C Mandell DJ Awareness in nine countries: a public health approach to suicide prevention Leg Med (Tokyo) 2009 11 Suppl 1 S13 17 19282224 10.1016/j.legalmed.2009.01.106

[B34] Zenere FJ Lazarus PJ The decline of youth suicidal behavior in an urban, multicultural public school system following the introduction of a suicide prevention and intervention program Suicide Life Threat Behav 1997 27 4 387 402 9444734

[B35] Reynolds TM Penney MD The mathematical basis of multivariate risk screening: with special reference to screening for Down's syndrome associated pregnancy Ann Clin Biochem 1990 27 Pt 5 452 458 2149257 10.1177/000456329002700506

[B36] Thompson EA Eggert LL Using the suicide risk screen to identify suicidal adolescents among potential high school dropouts J Am Acad Child Adolesc Psychiatry 1999 38 12 1506 1514 10.1097/00004583-199912000-00011 10596250

[B37] Bridge JA Goldstein TR Brent DA Adolescent suicide and suicidal behavior J Child Psychol Psychiatry 2006 47 3-4 372 394 10.1111/j.1469-7610.2006.01615.x 16492264

[B38] Mann JJ Apter A Bertolote J Beautrais A Currier D Haas A Hegerl U Lonnqvist J Malone K Marusic A Suicide prevention strategies: a systematic review JAMA 2005 294 16 2064 2074 10.1001/jama.294.16.2064 16249421

[B39] Rutz W von Knorring L Walinder J Long-term effects of an educational program for general practitioners given by the Swedish Committee for the Prevention and Treatment of Depression Acta Psychiatr Scand 1992 85 1 83 88 10.1111/j.1600-0447.1992.tb01448.x 1546555

[B40] Wasserman D Depression - the facts 2006 Oxford: Oxford University Press

[B41] Wasserman D (Ed) Suicide: an unnecessary death 2001 London: Martin Dunitz

[B42] World Health Organization Global School-Based Student Health Survey http://www.who.int/chp/gshs/en/

[B43] World Health Organization WHO Well-being scale (WHO-5) http://www.who-5.org/

[B44] Beck AT Steer RA Ball R Ranieri W Comparison of Beck Depression Inventories -IA and -II in psychiatric outpatients J Pers Assess 1996 67 3 588 597 10.1207/s15327752jpa6703_13 8991972

[B45] Paykel ES Myers JK Lindenthal JJ Tanner J Suicidal feelings in the general population: a prevalence study Br J Psychiatry 1972 124 380 406 10.1192/bjp.124.5.460 4836376

[B46] Goodman R Meltzer H Bailey V The Strengths and Difficulties Questionnaire: a pilot study on the validity of the self-report version Eur Child Adolesc Psychiatry 1998 7 3 125 130 10.1007/s007870050057 9826298

[B47] Gratz K Measurement of deliberate self-harm: preliminary data on the Deliberate Self-Harm Inventory Journal of Psychopathology and Behavioural Assessment 2001 23 253 263 10.1023/A:1012779403943

[B48] Young KS Psychology of computer use: XL. Addictive use of the Internet: a case that breaks the stereotype Psychol Rep 1996 79 3 Pt 1 899 902 8969098 10.2466/pr0.1996.79.3.899

[B49] European Values Study The European Values Study Questionnaire http://www.europeanvalues.nl/

[B50] Faden R Beauchamp T A History of Theory of Informed Consent 1986 New York: Oxford University Press

[B51] Reiter-Theil S Tröhler U, Reiter-Theil S Answers to Change: the Problem of Paradigm Shift in Medical Ethics from the German Standpoint Ethics Codes in Ethics from the German Standpoint Foundations and Achievements since 1947 1998 Ashgate: Aldershot 257 269

[B52] Wasserman D Wasserman C (Eds) The Oxford Textbook of Suicidology and Suicide Prevention: A Global Perspective 2009 1 Oxford: Oxford University Press

[B53] Wasserman C Hoven C Wasserman D Wasserman C, Hoven C, Wasserman D Affect and improve the way you feel 2009 Stockholm: Edita AB Avalaible languages: *English, Estonian, German, French, Hungarian, Hebrew, Italian, Romanian, Slovenian, Spanish*

[B54] Wasserman C Hoven C Wasserman D Wasserman C, Hoven C, Wasserman D Instructions for the school-based Awareness Intervention: Affect and improve the way you feel 2009 Stockholm: Edita AB Avalaible languages *: English, Estonian, German, French, Hungarian, Hebrew, Italian, Romanian, Slovenian, Spanish*

[B55] Beauchamp T Childress J Principles of Biomedical Ethics 1991 Oxford: Oxford University Press

[B56] Eich H Reiter L Reiter-Theil S Informed consent in psychotherapy: Singular action or continuing process? Ethical considerations about case study of child abuse Der Psychotherapeut 1997 42 369 375 10.1007/s002780050089

[B57] Cohen J Statistical Power Analysis for the Behavioral Sciences 1988 2 New Jersey: Lawrence Erlbaum

[B58] Höger C Reiter-Theil S Reiter L Derichs G Kastner-Voigt M Schulz T Ethical case reflection - a process model for ethics consultation in child psychiatry and psychotherapy System Familie 1997 10 174 179 10.1007/s004910050018

[B59] Teng EJ Friedman LC Increasing mental health awareness and appropriate service use in older Chinese Americans: a pilot intervention Patient Educ Couns 2009 76 1 143 146 10.1016/j.pec.2008.11.008 19124215

[B60] Garland AF Zigler E Adolescent suicide prevention. Current research and social policy implications Am Psychol 1993 48 2 169 182 10.1037/0003-066X.48.2.169 8442571

[B61] King KA Smith J Project SOAR: a training program to increase school counselors' knowledge and confidence regarding suicide prevention and intervention J Sch Health 2000 70 10 402 407 10.1111/j.1746-1561.2000.tb07227.x 11195950

[B62] Nelson FL Evaluation of a youth suicide prevention school program Adolescence 1987 22 88 813 825 3434398

[B63] Gould MS Brunstein-Klomek A Batejan K Wasserman D, Wasserman C The role of schools, colleges and universities in suicide prevention The Oxford Textbook of Suicidology and Suicide Prevention: A Global Perspective 2009 Oxford University Press 551 563

[B64] Reiter-Theil S Eich H Reiter L The ethical status of the child in family and child psychotherapy Praxis Kinderpsychol and Kinderpsychiat 1993 42 14 20 8441745

